# *catena*-Poly[[(6,7,9,10,17,18,20,21-octa­hydro-5,8,11,16,19,22-hexa­oxadibenzo[*a*,*j*]cyclo­octa­decene)barium]-di-μ-thio­cyanato-[thio­cyanato­diaurate(I)(*Au*—*Au*)]-μ-thio­cyanato]

**DOI:** 10.1107/S1600536809045218

**Published:** 2009-11-04

**Authors:** Tonia L. Stroud, Nathan L. Coker, Jeanette A. Krause

**Affiliations:** a Department of Physical Sciences, Morehead State University, Morehead, KY 40351, USA; b Department of Chemistry, University of Cincinnati, Cincinnati, OH 45221-0172, USA

## Abstract

In the title compound, [Au_2_Ba(NCS)_4_(C_20_H_24_O_6_)]_*n*_, the dithio­­cyanato­aurate(I) anion adopts a dimeric structure with an Au⋯Au distance of 3.1109 (10) Å; both Au^I^ atoms are also bonded to two S atoms. The Ba^II^ ion adopts an irregular BaN_3_O_6_ geometry, arising from the crown ether and three adjacent thio­cyanate N atoms; the extended structure of the complex can be described as a one-dimensional coordination polymer generated by the Ba⋯N inter­actions (two on the *endo* side and one on the *exo* side of the crown ether) running parallel to the *b* axis, with an anti­parallel arrangement of ribbons in the unit cell.

## Related literature

For further information on gold chemistry, see: Arvapally *et al.* (2007 ▶); Beavers *et al.* (2009 ▶); Chen *et al.* (2005 ▶); Coker (2003 ▶); Coker *et al.* (2004*a* ▶,*b* ▶, 2006 ▶); Mohamed *et al.* (2003 ▶); Olmstead *et al.* (2005 ▶); Pathaneni & Desiraju (1993 ▶); Schwerdtferger *et al.* (1990 ▶). For further information on barium macrocycles, see: Bordunov *et al.* (1996 ▶); Bradshaw & Izatt (1997 ▶); Felton *et al.* (2008 ▶); Henke & Atwood (1998 ▶); Masci & Thuery (2006 ▶); Metz *et al.* (1973 ▶). For a description of the Cambridge Structural Database, see: Allen (2002 ▶).
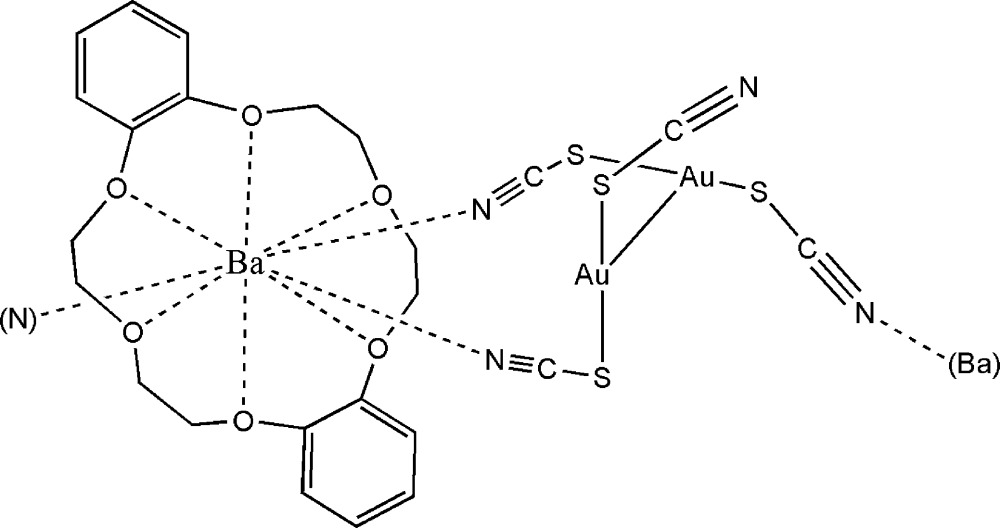


## Experimental

### 

#### Crystal data

[Au_2_Ba(NCS)_4_(C_20_H_24_O_6_)]
*M* *_r_* = 1123.98
Monoclinic, 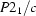

*a* = 17.5491 (8) Å
*b* = 12.6183 (4) Å
*c* = 15.6584 (6) Å
β = 110.598 (2)°
*V* = 3245.7 (2) Å^3^
*Z* = 4
Cu *K*α radiation
μ = 28.76 mm^−1^
*T* = 150 K
0.14 × 0.08 × 0.01 mm

#### Data collection

Bruker SMART6000 CCD diffractometer
Absorption correction: multi-scan (*SADABS*; Sheldrick, 2003 ▶) *T*_min_ = 0.118, *T*_max_ = 0.752
14223 measured reflections
4110 independent reflections
2905 reflections with *I* > 2σ(*I*)
*R*_int_ = 0.069
θ_max_ = 56.9°

#### Refinement

*R*[*F* ^2^ > 2σ(*F* ^2^)] = 0.053
*wR*(*F* ^2^) = 0.128
*S* = 0.98
4110 reflections
358 parameters
H-atom parameters constrained
Δρ_max_ = 1.66 e Å^−3^
Δρ_min_ = −0.85 e Å^−3^

### 

Data collection: *SMART* (Bruker, 2003 ▶); cell refinement: *SAINT* (Bruker, 2003 ▶); data reduction: *SAINT*; program(s) used to solve structure: *SHELXTL* (Sheldrick, 2008 ▶); program(s) used to refine structure: *SHELXTL*; molecular graphics: *SHELXTL*; software used to prepare material for publication: *SHELXTL*.

## Supplementary Material

Crystal structure: contains datablocks I, global. DOI: 10.1107/S1600536809045218/hb5102sup1.cif

Structure factors: contains datablocks I. DOI: 10.1107/S1600536809045218/hb5102Isup2.hkl

Additional supplementary materials:  crystallographic information; 3D view; checkCIF report

## Figures and Tables

**Table 1:** Selected bond lengths (Å)

Au1—S2	2.297 (4)
Au1—S1	2.305 (4)
Au2—S4	2.288 (4)
Au2—S3	2.305 (4)
Ba—N1	2.834 (14)
Ba—N2^i^	2.877 (13)
Ba—N3	2.774 (14)
Ba—O7	2.840 (9)
Ba—O17	2.845 (9)
Ba—O19	2.921 (12)
Ba—O9	2.927 (12)
Ba—O15	2.940 (11)
Ba—O5	2.988 (10)

Symmetry code: (i) 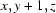.

**Fig. 1.**
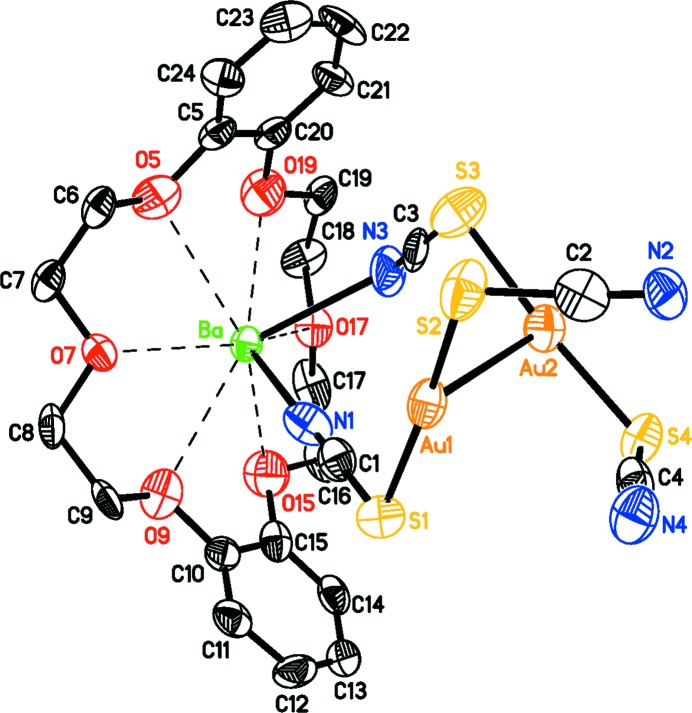
: Structure of (I) showing 50% probability ellipsoids. H-atoms omitted for clarity.

**Fig. 2.**
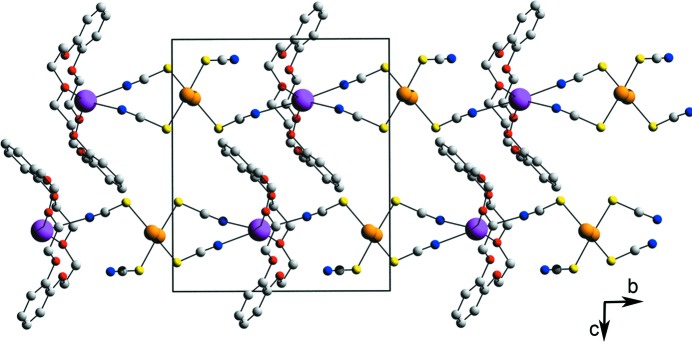
: The one-dimensional polymer chain runs parallel to the b axis, with an antiparallel arrangement of ribbons in the unit cell. H-atoms omitted for clarity.

## supplementary crystallographic information

### Comment

The present work stems from our interest in developing gold(I)-thiocyanate complexes with interesting gold bonding motifs and luminescent properties (Coker *et al.*, 2004*a*, Arvapally *et al.*,2007). We have shown that alkali (K^+^, Rb^+^ and Cs^+^) salts of bis(thiocyanato)aurate(I) (Coker *et al.*, 2004*a*) crystallize as linear one-dimensional polymeric chains with Au—Au distances in the 3.0065 (5)–3.2654 (2) Å range. Bis(thiocyanato)aurate(I) complexes with NH_4_^+^ (Coker *et al.*, 2006) and Me_4_N^+^ (Coker *et al.*, 2004*a*) adopt a similar linear or nearly linear motif, respectively, with alternating sets of Au—Au distances (NH_4_^+^: 3.1794 (2), 3.2654 (2) Å; Me_4_N^+^: 3.1409 (3), 3.1723 (3) Å). In sharp contrast, the anion in [(n-Bu)_4_N]bis(thiocyanato)aurate(I) (Coker *et al.*, 2004*a*) crystallizes as a dimer (Au—Au = 3.0700 (8) Å) while the anion in [Ph_4_As]bis(thiocyanato)aurate(I) (Schwerdtferger *et al.*, 1990) and [Ph_4_P]bis(thiocyanato)aurate(I) (Coker, 2003) exist as monomers.

To further explore the influence of the cation on the motif adopted by the bis(thiocyanato)aurate(I) anion, we synthesized and characterized the (1,4,7,10,13,16-hexaoxacyclooctadecane)-potassium dithiocyanatoaurate(I) [1,4,7,10,13,16-hexaoxacyclooctadecane = 18-crown-6] (Coker *et al.*, 2004*b*) and (18-crown-6)-caesium dithiocyanatoaurate(I) (Coker, 2003) complexes. The geometry of the [Au(SCN)_2_]^-^ anion in these complexes is monomeric and analogous to the Ph_4_P^+^ and Ph_4_As^+^ salts. The extended structure can be described as a zigzag polymeric chain formed by the coordination of the N atoms of the thiocyanate *via* a single intermolecular interaction to the vacant coordination site on the K or Cs atoms. To extend our bonding motif investigation, complexes utilizing 6,7,9,10,17,18,20,21-octahydro-5,8,11,16,19,22-hexaoxa- dibenzo[a,j]cyclooctadecene (dbz-18-crown-6) with alkali or alkaline earth cations were studied. In the present work, the structure of the title complex, (I), is reported.

The geometry of the anion in (I) (Fig. 1) is a dimer with a Au—Au bond distance of 3.1109 (10) Å and Au—S distances falling in the 2.299 (4)–2.305 (4) Å range. The Au—Au bond distance observed in (I) is less than the sum of the van der Waals radii of 3.32 Å for a gold-gold interaction (Bondi, 1964). A Cambridge Structural Database (CSD) analysis of gold-gold interactions reported by Pathaneni & Desiraju (1993) found that distances in the range 2.6–3.4 Å can be considered to have Au—Au bonding character. Furthermore, the Au—Au and Au—S bond distances in (I) are comparable to those in the related crown complex [CH_3_CN-(dbz-18-crown-6-Na)]_2_[Au(SCN)_2_]_2_.dbz-18-crown-6.CH_3_CN (Au—Au = 3.0661 (4) Å, Au—S = 2.291 (2)–2.303 (2) Å (Coker, 2003). The metallomacrocyclic gold(I) thiolate cluster, [Au_9_(µ-dppm)_4_(µ-*p*-tc)_6_](PF_6_)_3_ (dppm = bis(diphenylphosphine)methane and *p*-tc = *p*-thiocresolate), is reported to have four distinct gold environments. These environments consist of (*a*) Au—Au phosphine bridged single bonds (3.0084 (6)–3.1439 (6) Å, (*b*) Au—Au sulfiur bridged single bonds (2.9950 (7)–3.1632 (6) Å, (*c*) Au—Au non-bridged single bonds (3.0135 (7)–3.1825 (7) Å and (*d*) sulfur bridged Au···Au nonbonded interactions (3.7155 (8)–3.9571 (7) Å (Chen *et al.*, 2005). In the same vein, the PMe_3_ analog of the antiarthritic gold drug Auranofin, [(Me_3_PAu)_2_(µ-TATG)]NO_3_ (TATG = 2,3,4,6-tetraacetyl-1-thio-*D*-glucopyranosato) (Mohamed *et al.*, 2003) forms a tetranuclear gold cluster with Au—Au and Au—S distances in the 3.106 (7)–3.144 (12) Å and 2.334 (3)–2.355 (3) Å range, respectively. A CSD survey (Cambridge Structural Database v5.30) (Allen, 2002) of metal-thiocyanate complexes reveals an average M—S distance of 2.39 Å (*M* = Pt, Pd, Ag or Au), the Au—S distances in (I) are consistent with this observation.

Alkali and alkaline earth cations have a preferred tendency to bind in a way that high coordination numbers are achieved. This characteristic makes them useful in applications where coordination- flexible ligating agents are a necessity (*e.g*. sequestration) (Bradshaw & Izatt, 1997). The nine-coordinate Ba atom in (I) is bound to the six oxygen atoms of the dbz-18-crown-6 (Ba—O distances range: 2.940 (9)–2.988 (10) Å) and sits 0.769 (5) Å out of the plane generated by these atoms. The remainder of the coordination sites consists of two *endo* side and one *exo* side Ba···N interaction (2.774 (14)–2.877 (13) Å). The fourth thiocyanate moiety (N4) remains uncoordinated, the nearest nonbonded distance to Ba is 4.728 (15) Å. Thus the extended structure of (I) (Fig. 2) can be described as a one-dimensional coordination polymer generated by Ba···N intermolecular interactions running parallel to the *b* axis, with an overall antiparallel arrangement of ribbons in the unit cell.

In contrast, [poly[triaquatetra-µ-cyanido-tetracyanidobis(1,4,10,13- tetraoxa-7,16-diazacyclo-octadecane)dibarium(II)tetragold(I) crystallizes as a coordination polymer with the [Au(CN)_2_]^-^ anion in monomer, dimer and trimer environments while the barium atoms are bound to the diaza-18-crown-6 and solvent water molecules in nine and ten-coordinate geometries (Beavers *et al.*, 2009). Reported Ba···O and Ba···N distances for this gold-cyanato complex are 2.761 (2)–2.929 (2) Å and 2.867 (3)–2.959 (3) Å, respectively. In the case of *catena*-poly[[diaqua(1,4,7,10,13,16-hexaoxa-cyclooctadecane)- barium(II)]-µ-cyano-[dicyano-platinum(II)]-µ-cyano], the extended structure is an alternating chain of [18-crown-6-Ba]^2+^ and [Pt(CN)_4_]^2-^ ions bound through the N-atom of the cyano group to the Ba^2+^ ion (ten-coordinate geometry about Ba, Ba···N = 2.901 (2) Å and the average Ba—O_crown_ and Ba—O_water_ distances are reported as 2.858 (17) Å and 2.883 (13) Å) (Olmstead *et al.*, 2005). The Ba···O and Ba···N bonds in (I) are also consistent with those observed in structures such as *M*(TMTH_2_)_2_.nH_2_O (*M* = Ca, Sr, Ba and TMT=2,4,6-trimercaptotriazine) (Henke & Atwood, 1998) and Ba-containing macroethers, cryptands or lariat ethers, *e.g*. aqua-(7,16-bis((5-chloro-8-hydroxy-2-quinolinyl)methyl)-1,4,10,13- tetra-oxa-7,16-diazacyclo-octadecane)-barium dibromide (Bordunov *et al.*, 1996), aqua-thiocyanato-crypt(222)-barium thiocyanate (Metz *et al.*, 1973), bis(triethylammonium)diaqua- [2.2.2-crypt]-barium bis((*p*-*tert*-butyl-[3.1.3]tetrahomodioxa- calix[4[arene)-dioxo-uranium) pentahydrate (Masci & Thuery, 2006) and (8-propyl-18,21,26,29-tetraoxa-1,7,9,15,32,35-hexaazapenta-*cyclo* (13.8.8.4^3,13^.0^6,34^.0^10,33^)pentatriaconta-3,5,10,12,32,34- hexaene)-bis(perchlorato)-barium (Felton *et al.*, 2008.

### Experimental

Reaction of barium hydroxide (1 equiv) with ammonium thiocyanate (2 eqiv) in water results in the formation of barium thiocyanate with the release of ammonia gas.

Barium bis(thiocyanato)aurate(I) was prepared following the method described by Coker *et al.*, (2004*a*). (I) was prepared by the analogous method described for (18-crown-6-*K*)dithiocyanatoaurate(I) (Coker *et al.*, 2004*b*). Diffraction quality crystals were obtained from slow diffusion of acetonitrile-diethyl ether solution at -4 °C.

### Refinement

The H-atoms were placed in calculated positions (C_aromatic_—H = 0.95 Å, C_methylene_—H = 0.99 Å). The isotropic displacement parameters for all hydrogen atoms were defined as 1.2*U*_eq_ of the adjacent atom. The maximum residual electron-density peaks are located approximately 1 Å from the Au atoms.

### Crystal data

**Table:**

[Au_2_Ba(NCS)_4_(C_20_H_24_O_6_)]	*F*(000) = 2088
*M**_r_* = 1123.98	*D*_x_ = 2.300 Mg m^−^^3^
Monoclinic, *P*2_1_/*c*	Cu *K*α radiation, λ = 1.54178 Å
Hall symbol: -P 2ybc	Cell parameters from 4482 reflections
*a* = 17.5491 (8) Å	θ = 4.4–56.9°
*b* = 12.6183 (4) Å	µ = 28.76 mm^−^^1^
*c* = 15.6584 (6) Å	*T* = 150 K
β = 110.598 (2)°	Plate, pale pink
*V* = 3245.7 (2) Å^3^	0.14 × 0.08 × 0.01 mm
*Z* = 4	

### Data collection

**Table:**

Bruker SMART6000 CCD diffractometer	4110 independent reflections
Radiation source: fine-focus sealed tube	2905 reflections with *I* > 2σ(*I*)
graphite	*R*_int_ = 0.069
Detector resolution: 0.92 pixels mm^-1^	θ_max_ = 56.9°, θ_min_ = 2.7°
ω scans	*h* = −16→17
Absorption correction: multi-scan (*SADABS*; Sheldrick, 2003)	*k* = −11→13
*T*_min_ = 0.118, *T*_max_ = 0.752	*l* = −16→17
14223 measured reflections	

### Refinement

**Table:**

Refinement on *F*^2^	Primary atom site location: structure-invariant direct methods
Least-squares matrix: full	Secondary atom site location: difference Fourier map
*R*[*F*^2^ > 2σ(*F*^2^)] = 0.053	Hydrogen site location: inferred from neighbouring sites
*wR*(*F*^2^) = 0.128	H-atom parameters constrained
*S* = 0.98	*w* = 1/[σ^2^(*F*_o_^2^) + (0.0766*P*)^2^] where *P* = (*F*_o_^2^ + 2*F*_c_^2^)/3
4110 reflections	(Δ/σ)_max_ < 0.001
358 parameters	Δρ_max_ = 1.66 e Å^−^^3^
0 restraints	Δρ_min_ = −0.85 e Å^−^^3^

### Special details

**Table:**

Experimental. A suitable crystal was mounted in a Cryo-loop with paratone-N and immediately tranferred to the goniostat bathed in a cold stream.The final unit cell is obtained from the refinement of the XYZ weighted centroids of reflections above 20 σ(I). Note that the absorption correction parameters Tmin and Tmax also reflect beam corrections, *etc*. As a result, the numerical values for Tmin and Tmax may differ from expected values based solely absorption effects and crystal size.
Geometry. All e.s.d.'s (except the e.s.d. in the dihedral angle between two l.s. planes) are estimated using the full covariance matrix. The cell e.s.d.'s are taken into account individually in the estimation of e.s.d.'s in distances, angles and torsion angles; correlations between e.s.d.'s in cell parameters are only used when they are defined by crystal symmetry. An approximate (isotropic) treatment of cell e.s.d.'s is used for estimating e.s.d.'s involving l.s. planes.
Refinement. Refinement of *F*^2^ against ALL reflections. The weighted *R*-factor *wR* and goodness of fit *S* are based on *F*^2^, conventional *R*-factors *R* are based on *F*, with *F* set to zero for negative *F*^2^. The threshold expression of *F*^2^ > σ(*F*^2^) is used only for calculating *R*-factors(gt) *etc*. and is not relevant to the choice of reflections for refinement. *R*-factors based on *F*^2^ are statistically about twice as large as those based on *F*, and *R*-factors based on ALL data will be even larger.The anisotropic displacement parameters for C7 and C20 were constrained to be equivalent to C6 and C5, respectively.

### Fractional atomic coordinates and isotropic or equivalent isotropic displacement parameters (Å^2^)

**Table:**

	*x*	*y*	*z*	*U*_iso_*/*U*_eq_	
Au1	0.34634 (4)	0.40337 (5)	0.26591 (4)	0.0408 (2)	
Au2	0.17476 (4)	0.43183 (5)	0.27917 (5)	0.0453 (3)	
S1	0.4056 (2)	0.5186 (3)	0.3851 (2)	0.0433 (11)	
S2	0.3048 (3)	0.2863 (3)	0.1460 (3)	0.0584 (13)	
S3	0.1241 (3)	0.5265 (4)	0.1450 (3)	0.0635 (14)	
S4	0.2067 (3)	0.3454 (3)	0.4158 (3)	0.0490 (11)	
C1	0.3550 (10)	0.6284 (13)	0.3434 (10)	0.041 (4)	
C2	0.2600 (10)	0.1899 (13)	0.1840 (11)	0.047 (4)	
C3	0.1250 (9)	0.6475 (14)	0.1921 (11)	0.047 (4)	
C4	0.2867 (11)	0.2679 (14)	0.4179 (11)	0.048 (5)	
N1	0.3197 (8)	0.7081 (11)	0.3177 (9)	0.051 (4)	
N2	0.2288 (8)	0.1179 (10)	0.2044 (9)	0.047 (4)	
N3	0.1230 (9)	0.7317 (11)	0.2215 (10)	0.060 (4)	
N4	0.3382 (9)	0.2131 (12)	0.4199 (10)	0.058 (4)	
Ba	0.23237 (6)	0.89893 (6)	0.25677 (6)	0.0322 (3)	
O5	0.1978 (8)	0.9218 (9)	0.0567 (7)	0.066 (4)	
O7	0.3582 (6)	0.9286 (7)	0.1843 (6)	0.036 (2)	
O9	0.3953 (7)	0.9610 (9)	0.3779 (7)	0.064 (3)	
O15	0.2715 (8)	0.9925 (10)	0.4392 (8)	0.065 (3)	
O17	0.1095 (6)	0.9937 (7)	0.3111 (6)	0.039 (3)	
O19	0.0748 (7)	0.9545 (9)	0.1208 (7)	0.064 (3)	
C5	0.1207 (10)	0.8637 (12)	0.0123 (9)	0.037 (3)	
C6	0.2689 (9)	0.8954 (11)	0.0301 (9)	0.036 (3)	
H6A	0.2800	0.8184	0.0371	0.043*	
H6B	0.2573	0.9146	−0.0346	0.043*	
C7	0.3402 (9)	0.9553 (11)	0.0894 (8)	0.036 (3)	
H7B	0.3880	0.9389	0.0721	0.043*	
H7A	0.3292	1.0322	0.0806	0.043*	
C8	0.4339 (9)	0.9751 (12)	0.2431 (9)	0.041 (4)	
H8A	0.4274	1.0527	0.2464	0.049*	
H8B	0.4773	0.9614	0.2177	0.049*	
C9	0.4576 (9)	0.9277 (12)	0.3375 (9)	0.040 (4)	
H9A	0.4594	0.8495	0.3339	0.048*	
H9B	0.5122	0.9532	0.3761	0.048*	
C10	0.4040 (10)	0.9142 (11)	0.4657 (9)	0.038 (4)	
C11	0.4680 (10)	0.8473 (11)	0.5139 (10)	0.040 (4)	
H11	0.5101	0.8316	0.4908	0.048*	
C12	0.4694 (11)	0.8042 (12)	0.5956 (10)	0.047 (4)	
H12	0.5129	0.7589	0.6291	0.057*	
C13	0.4087 (11)	0.8263 (12)	0.6286 (11)	0.044 (4)	
H13	0.4112	0.7978	0.6857	0.053*	
C14	0.3448 (10)	0.8886 (11)	0.5806 (10)	0.040 (4)	
H14	0.3029	0.9013	0.6046	0.049*	
C15	0.3385 (11)	0.9342 (11)	0.4980 (10)	0.037 (4)	
C16	0.2001 (10)	1.0007 (13)	0.4669 (11)	0.050 (5)	
H16B	0.2145	1.0419	0.5242	0.061*	
H16A	0.1833	0.9289	0.4787	0.061*	
C17	0.1299 (11)	1.0539 (13)	0.3941 (12)	0.056 (5)	
H17A	0.0822	1.0578	0.4139	0.067*	
H17B	0.1453	1.1270	0.3838	0.067*	
C18	0.0412 (10)	1.0419 (12)	0.2411 (10)	0.048 (5)	
H18B	0.0578	1.1098	0.2212	0.058*	
H18A	−0.0032	1.0562	0.2647	0.058*	
C19	0.0129 (9)	0.9649 (13)	0.1622 (10)	0.042 (4)	
H19B	0.0023	0.8948	0.1843	0.050*	
H19A	−0.0384	0.9906	0.1162	0.050*	
C20	0.0582 (10)	0.8804 (11)	0.0468 (9)	0.037 (3)	
C21	−0.0125 (10)	0.8211 (12)	0.0105 (10)	0.045 (4)	
H21	−0.0565	0.8328	0.0313	0.054*	
C22	−0.0195 (10)	0.7466 (12)	−0.0542 (11)	0.054 (5)	
H22	−0.0671	0.7039	−0.0758	0.064*	
C23	0.0427 (12)	0.7322 (13)	−0.0892 (11)	0.051 (5)	
H23	0.0359	0.6824	−0.1368	0.061*	
C24	0.1134 (10)	0.7894 (12)	−0.0554 (10)	0.040 (4)	
H24	0.1565	0.7784	−0.0779	0.049*	

### Atomic displacement parameters (Å^2^)

**Table:**

	*U*^11^	*U*^22^	*U*^33^	*U*^12^	*U*^13^	*U*^23^
Au1	0.0510 (5)	0.0316 (4)	0.0483 (4)	0.0031 (3)	0.0280 (4)	0.0028 (3)
Au2	0.0490 (5)	0.0377 (4)	0.0535 (4)	0.0027 (4)	0.0235 (4)	0.0008 (3)
S1	0.044 (3)	0.043 (2)	0.042 (2)	0.010 (2)	0.015 (2)	−0.0041 (19)
S2	0.099 (4)	0.039 (2)	0.053 (2)	−0.007 (2)	0.046 (3)	−0.005 (2)
S3	0.079 (4)	0.069 (3)	0.049 (2)	0.022 (3)	0.030 (3)	0.008 (2)
S4	0.063 (3)	0.039 (2)	0.054 (2)	0.009 (2)	0.033 (2)	0.009 (2)
C1	0.037 (11)	0.042 (9)	0.048 (9)	−0.002 (9)	0.021 (9)	−0.004 (8)
C2	0.045 (12)	0.044 (10)	0.056 (10)	0.011 (9)	0.022 (10)	−0.010 (9)
C3	0.031 (11)	0.056 (11)	0.056 (10)	−0.002 (9)	0.018 (9)	0.034 (10)
C4	0.059 (14)	0.051 (11)	0.048 (10)	−0.022 (10)	0.035 (11)	−0.014 (9)
N1	0.040 (10)	0.050 (9)	0.059 (8)	0.000 (8)	0.012 (8)	−0.014 (7)
N2	0.060 (11)	0.028 (7)	0.053 (8)	−0.008 (7)	0.018 (8)	−0.006 (6)
N3	0.065 (11)	0.035 (8)	0.079 (10)	−0.007 (8)	0.024 (9)	0.017 (8)
N4	0.064 (12)	0.059 (10)	0.064 (9)	0.006 (9)	0.037 (9)	0.008 (8)
Ba	0.0354 (6)	0.0300 (5)	0.0345 (5)	0.0007 (4)	0.0166 (4)	0.0001 (4)
O5	0.074 (10)	0.077 (9)	0.045 (6)	−0.001 (7)	0.021 (7)	−0.001 (6)
O7	0.030 (6)	0.039 (6)	0.041 (5)	−0.004 (5)	0.013 (5)	−0.003 (5)
O9	0.073 (9)	0.063 (8)	0.064 (7)	−0.001 (7)	0.033 (7)	0.007 (6)
O15	0.057 (9)	0.081 (9)	0.065 (7)	0.004 (7)	0.031 (7)	−0.004 (7)
O17	0.050 (7)	0.032 (5)	0.046 (6)	0.006 (5)	0.029 (6)	−0.011 (5)
O19	0.069 (9)	0.073 (8)	0.060 (7)	0.000 (7)	0.035 (7)	−0.007 (7)
C5	0.039 (9)	0.042 (6)	0.028 (6)	0.009 (6)	0.010 (5)	0.011 (5)
C6	0.040 (8)	0.042 (6)	0.036 (6)	0.007 (5)	0.028 (5)	0.003 (5)
C7	0.040 (8)	0.042 (6)	0.036 (6)	0.007 (5)	0.028 (5)	0.003 (5)
C8	0.047 (12)	0.041 (9)	0.046 (9)	−0.018 (8)	0.030 (9)	−0.005 (8)
C9	0.023 (10)	0.053 (10)	0.047 (9)	−0.018 (8)	0.018 (8)	−0.012 (8)
C10	0.041 (11)	0.040 (9)	0.032 (8)	−0.009 (8)	0.011 (8)	−0.006 (8)
C11	0.039 (11)	0.028 (8)	0.048 (9)	−0.007 (8)	0.008 (9)	−0.008 (8)
C12	0.049 (12)	0.036 (9)	0.043 (9)	0.008 (8)	0.000 (9)	0.011 (8)
C13	0.044 (12)	0.042 (10)	0.043 (9)	−0.011 (9)	0.010 (10)	0.004 (8)
C14	0.044 (12)	0.038 (9)	0.044 (9)	−0.018 (9)	0.020 (9)	−0.014 (8)
C15	0.048 (12)	0.027 (8)	0.043 (9)	−0.010 (8)	0.025 (9)	−0.013 (7)
C16	0.063 (14)	0.047 (10)	0.058 (11)	−0.012 (10)	0.043 (11)	−0.033 (9)
C17	0.051 (13)	0.043 (10)	0.094 (14)	−0.001 (9)	0.050 (12)	−0.020 (10)
C18	0.055 (12)	0.033 (9)	0.053 (10)	0.029 (8)	0.014 (9)	0.010 (8)
C19	0.036 (11)	0.058 (10)	0.043 (9)	0.008 (8)	0.028 (9)	0.011 (8)
C20	0.039 (9)	0.042 (6)	0.028 (6)	0.009 (6)	0.010 (5)	0.011 (5)
C21	0.030 (11)	0.038 (9)	0.052 (9)	−0.003 (8)	−0.003 (9)	0.000 (8)
C22	0.023 (11)	0.036 (10)	0.075 (12)	−0.003 (8)	−0.016 (10)	0.000 (9)
C23	0.050 (13)	0.046 (10)	0.048 (10)	0.026 (10)	0.006 (10)	0.013 (9)
C24	0.028 (11)	0.045 (9)	0.046 (9)	0.009 (8)	0.010 (9)	−0.001 (8)

### Geometric parameters (Å, °)

**Table:**

Au1—S2	2.297 (4)	C6—H6A	0.9900
Au1—S1	2.305 (4)	C6—H6B	0.9900
Au1—Au2	3.1109 (10)	C7—H7B	0.9900
Au2—S4	2.288 (4)	C7—H7A	0.9900
Au2—S3	2.305 (4)	C8—C9	1.510 (18)
S1—C1	1.651 (17)	C8—H8A	0.9900
S2—C2	1.668 (19)	C8—H8B	0.9900
S3—C3	1.69 (2)	C9—H9A	0.9900
S4—C4	1.70 (2)	C9—H9B	0.9900
C1—N1	1.176 (18)	C10—C11	1.39 (2)
C2—N2	1.162 (19)	C10—C15	1.43 (2)
C3—N3	1.16 (2)	C11—C12	1.38 (2)
C4—N4	1.131 (19)	C11—H11	0.9500
N2—Ba^i^	2.877 (13)	C12—C13	1.37 (2)
Ba—N1	2.834 (14)	C12—H12	0.9500
Ba—N2^ii^	2.877 (13)	C13—C14	1.36 (2)
Ba—N3	2.774 (14)	C13—H13	0.9500
Ba—O7	2.840 (9)	C14—C15	1.38 (2)
Ba—O17	2.845 (9)	C14—H14	0.9500
Ba—O19	2.921 (12)	C16—C17	1.51 (2)
Ba—O9	2.927 (12)	C16—H16B	0.9900
Ba—O15	2.940 (11)	C16—H16A	0.9900
Ba—O5	2.988 (10)	C17—H17A	0.9900
O5—C5	1.479 (18)	C17—H17B	0.9900
O5—C6	1.486 (17)	C18—C19	1.51 (2)
O7—C7	1.446 (15)	C18—H18B	0.9900
O7—C8	1.447 (16)	C18—H18A	0.9900
O9—C10	1.454 (17)	C19—H19B	0.9900
O9—C9	1.504 (18)	C19—H19A	0.9900
O15—C15	1.418 (18)	C20—C21	1.39 (2)
O15—C16	1.467 (18)	C21—C22	1.35 (2)
O17—C17	1.438 (17)	C21—H21	0.9500
O17—C18	1.443 (16)	C22—C23	1.39 (2)
O19—C20	1.436 (18)	C22—H22	0.9500
O19—C19	1.453 (17)	C23—C24	1.37 (2)
C5—C24	1.388 (19)	C23—H23	0.9500
C5—C20	1.40 (2)	C24—H24	0.9500
C6—C7	1.477 (19)		
			
S2—Au1—S1	172.10 (16)	O5—C6—H6B	110.0
S2—Au1—Au2	95.32 (13)	H6A—C6—H6B	108.4
S1—Au1—Au2	92.58 (10)	O7—C7—C6	111.0 (11)
S4—Au2—S3	171.03 (16)	O7—C7—H7B	109.4
S4—Au2—Au1	94.73 (11)	C6—C7—H7B	109.4
S3—Au2—Au1	94.10 (12)	O7—C7—H7A	109.4
C1—S1—Au1	100.5 (5)	C6—C7—H7A	109.4
C2—S2—Au1	103.4 (5)	H7B—C7—H7A	108.0
C3—S3—Au2	97.5 (5)	O7—C8—C9	109.7 (11)
C4—S4—Au2	102.8 (6)	O7—C8—H8A	109.7
N1—C1—S1	177.0 (14)	C9—C8—H8A	109.7
N2—C2—S2	174.5 (14)	O7—C8—H8B	109.7
N3—C3—S3	177.4 (16)	C9—C8—H8B	109.7
N4—C4—S4	177.4 (16)	H8A—C8—H8B	108.2
C1—N1—Ba	179.0 (13)	O9—C9—C8	108.1 (12)
C2—N2—Ba^i^	149.7 (12)	O9—C9—H9A	110.1
C3—N3—Ba	131.2 (13)	C8—C9—H9A	110.1
N3—Ba—N1	71.0 (4)	O9—C9—H9B	110.1
N3—Ba—O7	127.0 (4)	C8—C9—H9B	110.1
N1—Ba—O7	80.9 (3)	H9A—C9—H9B	108.4
N3—Ba—O17	80.5 (4)	C11—C10—C15	120.6 (14)
N1—Ba—O17	129.3 (3)	C11—C10—O9	123.7 (14)
O7—Ba—O17	147.3 (3)	C15—C10—O9	115.6 (13)
N3—Ba—N2^ii^	136.9 (4)	C12—C11—C10	119.2 (15)
N1—Ba—N2^ii^	150.2 (4)	C12—C11—H11	120.4
O7—Ba—N2^ii^	72.6 (3)	C10—C11—H11	120.4
O17—Ba—N2^ii^	74.8 (3)	C13—C12—C11	120.5 (15)
N3—Ba—O19	68.0 (4)	C13—C12—H12	119.8
N1—Ba—O19	135.3 (3)	C11—C12—H12	119.8
O7—Ba—O19	110.3 (3)	C14—C13—C12	120.7 (15)
O17—Ba—O19	59.6 (3)	C14—C13—H13	119.6
N2^ii^—Ba—O19	69.0 (4)	C12—C13—H13	119.6
N3—Ba—O9	141.5 (4)	C13—C14—C15	122.4 (16)
N1—Ba—O9	73.8 (3)	C13—C14—H14	118.8
O7—Ba—O9	60.3 (3)	C15—C14—H14	118.8
O17—Ba—O9	111.3 (3)	C14—C15—O15	126.7 (15)
N2^ii^—Ba—O9	81.1 (3)	C14—C15—C10	116.6 (15)
O19—Ba—O9	150.1 (3)	O15—C15—C10	116.6 (13)
N3—Ba—O15	114.1 (4)	O15—C16—C17	111.3 (13)
N1—Ba—O15	95.8 (4)	O15—C16—H16B	109.4
O7—Ba—O15	112.7 (3)	C17—C16—H16B	109.4
O17—Ba—O15	58.9 (3)	O15—C16—H16A	109.4
N2^ii^—Ba—O15	82.3 (3)	C17—C16—H16A	109.4
O19—Ba—O15	116.7 (3)	H16B—C16—H16A	108.0
O9—Ba—O15	54.7 (3)	O17—C17—C16	108.9 (12)
N3—Ba—O5	89.6 (4)	O17—C17—H17A	109.9
N1—Ba—O5	108.7 (4)	C16—C17—H17A	109.9
O7—Ba—O5	57.9 (3)	O17—C17—H17B	109.9
O17—Ba—O5	112.1 (3)	C16—C17—H17B	109.9
N2^ii^—Ba—O5	68.4 (3)	H17A—C17—H17B	108.3
O19—Ba—O5	54.6 (3)	O17—C18—C19	107.2 (11)
O9—Ba—O5	116.4 (3)	O17—C18—H18B	110.3
O15—Ba—O5	150.7 (3)	C19—C18—H18B	110.3
C5—O5—C6	118.4 (11)	O17—C18—H18A	110.3
C5—O5—Ba	105.1 (8)	C19—C18—H18A	110.3
C6—O5—Ba	113.0 (8)	H18B—C18—H18A	108.5
C7—O7—C8	112.3 (10)	O19—C19—C18	109.9 (13)
C7—O7—Ba	121.4 (8)	O19—C19—H19B	109.7
C8—O7—Ba	117.9 (7)	C18—C19—H19B	109.7
C10—O9—C9	115.7 (12)	O19—C19—H19A	109.7
C10—O9—Ba	105.0 (8)	C18—C19—H19A	109.7
C9—O9—Ba	110.0 (8)	H19B—C19—H19A	108.2
C15—O15—C16	116.4 (12)	C21—C20—C5	118.0 (14)
C15—O15—Ba	106.3 (8)	C21—C20—O19	125.5 (14)
C16—O15—Ba	112.6 (8)	C5—C20—O19	116.4 (14)
C17—O17—C18	109.8 (11)	C22—C21—C20	120.9 (16)
C17—O17—Ba	121.3 (9)	C22—C21—H21	119.6
C18—O17—Ba	117.7 (8)	C20—C21—H21	119.6
C20—O19—C19	116.6 (12)	C21—C22—C23	120.4 (16)
C20—O19—Ba	107.6 (8)	C21—C22—H22	119.8
C19—O19—Ba	111.1 (8)	C23—C22—H22	119.8
C24—C5—C20	121.6 (15)	C24—C23—C22	120.5 (16)
C24—C5—O5	121.1 (14)	C24—C23—H23	119.7
C20—C5—O5	117.1 (13)	C22—C23—H23	119.7
C7—C6—O5	108.6 (11)	C23—C24—C5	118.5 (16)
C7—C6—H6A	110.0	C23—C24—H24	120.7
O5—C6—H6A	110.0	C5—C24—H24	120.7
C7—C6—H6B	110.0		
			
S2—Au1—Au2—S4	110.83 (15)	C10—C11—C12—C13	0(2)
S1—Au1—Au2—S4	−69.26 (14)	C11—C12—C13—C14	2(2)
S2—Au1—Au2—S3	−70.74 (16)	C12—C13—C14—C15	−1(2)
S1—Au1—Au2—S3	109.17 (16)	C13—C14—C15—O15	174.1 (13)
S2—Au1—S1—C1	118.4 (11)	C13—C14—C15—C10	−1(2)
Au2—Au1—S1—C1	−60.9 (6)	C16—O15—C15—C14	−3(2)
S1—Au1—S2—C2	125.3 (11)	C16—O15—C15—C10	172.1 (12)
Au2—Au1—S2—C2	−55.4 (6)	C11—C10—C15—C14	3(2)
S4—Au2—S3—C3	64.5 (13)	O9—C10—C15—C14	178.7 (12)
Au1—Au2—S3—C3	−105.4 (6)	C11—C10—C15—O15	−172.5 (12)
S3—Au2—S4—C4	167.1 (11)	O9—C10—C15—O15	3.0 (18)
Au1—Au2—S4—C4	−23.0 (6)	C15—O15—C16—C17	−173.8 (12)
Au1—S1—C1—N1	154 (30)	C18—O17—C17—C16	178.7 (13)
Au1—S2—C2—N2	−168 (17)	O15—C16—C17—O17	58.9 (16)
Au2—S3—C3—N3	−148 (35)	C17—O17—C18—C19	−169.6 (12)
Au2—S4—C4—N4	−125 (37)	C20—O19—C19—C18	177.5 (11)
S1—C1—N1—Ba	−68 (93)	O17—C18—C19—O19	−67.0 (15)
S2—C2—N2—Ba^i^	61 (18)	C24—C5—C20—C21	−1(2)
S3—C3—N3—Ba	−115 (35)	O5—C5—C20—C21	−175.5 (12)
C6—O5—C5—C24	−2.7 (18)	C24—C5—C20—O19	176.2 (12)
C6—O5—C5—C20	172.0 (12)	O5—C5—C20—O19	1.4 (18)
C5—O5—C6—C7	−174.4 (11)	C19—O19—C20—C21	2(2)
C8—O7—C7—C6	172.3 (11)	C19—O19—C20—C5	−174.6 (12)
O5—C6—C7—O7	59.6 (14)	C5—C20—C21—C22	2(2)
C7—O7—C8—C9	−169.0 (11)	O19—C20—C21—C22	−174.3 (13)
C10—O9—C9—C8	174.3 (11)	C20—C21—C22—C23	−4(2)
O7—C8—C9—O9	−66.0 (14)	C21—C22—C23—C24	3(2)
C9—O9—C10—C11	3.7 (19)	C22—C23—C24—C5	−2(2)
C9—O9—C10—C15	−171.6 (12)	C20—C5—C24—C23	1(2)
C15—C10—C11—C12	−3(2)	O5—C5—C24—C23	175.1 (12)
O9—C10—C11—C12	−178.0 (13)		

Symmetry codes: (i) *x*, *y*−1, *z*; (ii) *x*, *y*+1, *z*.
